# Noninvasive Fetal Electrocardiography Part II: Segmented-Beat Modulation Method for Signal Denoising

**DOI:** 10.2174/1874120701711010025

**Published:** 2017-03-31

**Authors:** Angela Agostinelli, Agnese Sbrollini, Luca Burattini, Sandro Fioretti, Francesco Di Nardo, Laura Burattini

**Affiliations:** 1 Department of Information Engineering, Università Politecnica delle Marche, Ancona, Italy; 2 Department of Clinical Sciences, Università Politecnica delle Marche, Ancona, Italy

**Keywords:** Abdominal fetal electrocardiography, Direct fetal electrocardiography, Digital electrocardiography, Fetal monitoring, Segmented-beat modulation method, Nonlinear filtering

## Abstract

**Background:**

Fetal well-being evaluation may be accomplished by monitoring cardiac activity through fetal electrocardiography. Direct fetal electrocardiography (acquired through scalp electrodes) is the gold standard but its invasiveness limits its clinical applicability. Instead, clinical use of indirect fetal electrocardiography (acquired through abdominal electrodes) is limited by its poor signal quality.

**Objective:**

Aim of this study was to evaluate the suitability of the Segmented-Beat Modulation Method to denoise indirect fetal electrocardiograms in order to achieve a signal-quality at least comparable to the direct ones.

**Method:**

Direct and indirect recordings, simultaneously acquired from 5 pregnant women during labor, were filtered with the Segmented-Beat Modulation Method and correlated in order to assess their morphological correspondence. Signal-to-noise ratio was used to quantify their quality.

**Results:**

Amplitude was higher in direct than indirect fetal electrocardiograms (median:104 µV *vs.* 22 µV; P=7.66·10^-4^), whereas noise was comparable (median:70 µV *vs.* 49 µV, P=0.45). Moreover, fetal electrocardiogram amplitude was significantly higher than affecting noise in direct recording (P=3.17·10^-2^) and significantly in indirect recording (P=1.90·10^-3^). Consequently, signal-to-noise ratio was initially higher for direct than indirect recordings (median:3.3 dB *vs.* -2.3 dB; P=3.90·10^-3^), but became lower after denoising of indirect ones (median:9.6 dB; P=9.84·10^-4^). Eventually, direct and indirect recordings were highly correlated (median: ρ=0.78; P<10^-208^), indicating that the two electrocardiograms were morphologically equivalent.

**Conclusion:**

Segmented-Beat Modulation Method is particularly useful for denoising of indirect fetal electrocardiogram and may contribute to the spread of this noninvasive technique in the clinical practice.

## 1 INTRODUCTION

Cardiac complications represent a common cause of birth death [1-3]. Fetal well-being evaluation is usually accomplished through cardiotocography, which provides information about the fetal status based on the fetal cardiac rhythm [4]. To reduce the number of operative deliveries possibly due to fetal distress, cardiotocography may be combined to fetal electrocardiography (FECG) [5-7] that, in addition to heart-rate monitoring, allows evaluations strictly related to the FECG morphology. Indeed, both P-wave and QRS-complex durations may be used to assess intrauterine growth restriction [8]; fetal supraventricular extrasystoles may indicate cases of congenital heart diseases to be treated during pregnancy or immediately after birth [9, 10]; eventually, fetal ST changes may indirectly indicate myocardial hypoxia [11, 12]. Direct FECG (DFECG), obtained from recordings acquired by directly positioning an electrode on the fetal scalp, is considered the gold standard but its invasiveness and its restricted applicability to the stage of labor only, have limited its clinical use [13]. Indirect FECG (IFECG), extracted from recordings acquired by positioning electrodes on the maternal abdomen [11, 14-16], is noninvasive and has a wider applicability also extended to the final weeks (approximately from the 38^th^, when the vernix caseosa, which almost electrically shields the fetus, start to dissolve) of gestation. Besides IFECG, such recordings typically contain maternal electrocardiogram, maternal and fetal muscular noise, fetal electroencephalography, and other kinds of noise [11, 15, 16]. Denoising these recordings for IFECG extraction is a very challenging task [14]; actually, IFECG clinical use is mainly limited by its poor signal quality. This work, which is the second of a two-paper series on noninvasive fetal electrocardiography [17], proposes a procedure for denoising indirect recordings in order to obtain an IFECG tracing characterized by a signal quality at least comparable to that of DFECG. Availability of such procedure would indeed justify use of IFECG instead of DFECG and could contribute to the clinical spread of FECG.

The Segmented-Beat Modulation Method (SBMM) was recently proposed [18-21] as a denoising technique for electrocardiograms. SBMM works under the hypothesis of knowing R peaks and its theoretic principles make it particularly suitable for FECG applications. We have proposed an adaptation of the Pan-Tompkins algorithm [22] to fetal R-peak detection [17]. Here, we evaluated SBMM suitability to denoise indirect recordings in order to obtain a good-quality IFECG tracings. To this aim, SBMM was applied to direct and indirect recordings simultaneously acquired from pregnant women during labor.

## 2 MATERIAL AND METHODS

### 2.1 Clinical Data and Data Modeling

Our clinical data (same as in [17]) consisted of 5 records 60 s long from 5 different pregnant women during labor, which occurred within the 38^th^-41^st^ week of gestation. The records were acquired from the Department of Obstetrics at the Medical University of Silesia, by means of the KOMPOREL system (sampling rate: 1000 Hz; resolution:16 bits) for acquisition and analysis of FECG (ITAM Institute, Zabrze, Poland). Each record was constituted by a direct recording (DREC) and a 4-channel indirect recording (IREC) simultaneously acquired. DREC was carried out with a spiral electrode on the fetal head; instead, IREC was obtained by placing 4 electrodes around the navel, a reference electrode above the pubic symphysis and a common mode reference electrode (with active-ground signal) on the left leg. All recordings are part of the “Abdominal and Direct Fetal Electrocardiogram Database” [23] of PhysioNet (www.physionet.org) [24], freely accessible on the web under the ODC Public Domain Dedication and License v1.0. The database has been fully anonymized and may be used without further Institutional Review Boards approval. Reference R-peak positions are also available; the R-wave locations were automatically determined in the direct FECG signal by means of on-line analysis applied in the KOMPOREL system. These locations were then verified (off-line) by a group of cardiologists, resulting in a set of reference markers precisely indicating the R-wave locations.

Given the acquisition modalities, DREC is substantially a noisy version of DFECG, whereas IREC, besides IFECG, also contains maternal electrocardiogram (MECG) and other noise kinds. Noise affecting DREC and IREC is a mixture of interferences that can or cannot have a physiological origin. It can be decomposed in low-frequency noise, high-frequency noise and in-band noise. The low-frequency noise is characterized by a frequency band between 0 and 0.5 Hz, where no electrocardiographic components (either fetal or maternal) are expected to fall. The high-frequency noise includes interferences that are characterized by frequency components above 45 Hz, where no significant electrocardiographic components (either fetal or maternal) are expected to fall. Eventually, the in-band noise has frequency components that overlap to the electrocardiographic ones (*i.e.* between 0.5 and 45 Hz). Before performing FECG extraction, DREC and IREC are prefiltered by application of a bandpass filter with cut-off frequencies of 0.5 Hz and 45 Hz [11]. Prefiltering allows removal of the low-frequency noise and the high-frequency noise. As a result, DREC and IREC can be mathematically modeled as follows:

(1) DREC=DFECG+DN

(2) IREC=IFECG+MECG+IN,

where DN e IN are the in-band noise components affecting DREC and IREC, respectively.

### 2.2 FECG Extraction

DFECG and IFECG were extracted from DREC and IREC, respectively, by application of SBMM (Fig. (**1**)). SBMM is a denoising procedure for electrocardiographic signals that works under the hypothesis of knowing R-peak positions [18-21]. A brief description of this method is reported in Appendix. All processing procedures were performed in Matlab using an SBMM implementation provided by B.M.E.D. Srl (Bio-Medical Engineering Development SRL, Ancona, Italy; www.bmed-bioengineering.com).

The block diagram representing DFECG extraction from DREC is depicted in Fig. (**1**), panel a. Then, reference fetal R-peak positions and DREC were submitted to SBMM, which provides DFECG as output. DN was obtained by subtracting DFECG from DREC.

SBMM was also used to extract an IFECG signal from each IREC channel (Fig. (**1**), panel b). Being MECG the highest amplitude component in IREC, maternal R peaks were obtained by applying the Pan-Tompkins algorithm [22] to IREC. Then, maternal R-peak positions and IREC were submitted to SBMM in order to get MECG. Successively, MECG was subtracted from IREC to obtain a noisy version of IFECG (IFECG+IN) which, together with fetal R-peak positions (which were the same used for DFECG extraction), was submitted to SBMM. Eventually, SBMM provided IFECG as output, whereas IN was obtained by subtraction.

### 2.3 Computation of the Signal-to-Noise Ratio Characterizing a Fetal Electrocardiogram

The Signal-to-Noise Ratio (SNR) is a useful parameter to relatively quantify the level of noise affecting a signal. Typically expressed in decibel (dB), it may be obtained as the ratio between the signal amplitude over the noise amplitude [25]. In our study, the signals of interest were DFECG and IFECG, respectively affected by DN and IN. Consequently, direct SNR (DSNR) and indirect SNR (ISNR) were obtained as follows:

(3) $$\mathrm{DSNR}=10\log_{10}\frac{\mathrm{DFECG}\;\mathrm{amplitude}}{\mathrm{DN}\;\mathrm{amplitude}}$$

(4) $$\mathrm{ISNR}=10\log_{10}\frac{\mathrm{IFECG}\;\mathrm{amplitude}}{\mathrm{IN}\;\mathrm{amplitude}}.$$

Being DFECG and IFECG close to deterministic (pseudo-periodic) signals, their amplitudes were computed as mean of the maximum-minus-minimum values over the beats. Instead, being DN and IN close to Gaussian stochastic signals, their amplitudes were computed as 4 times standard deviation [26, 27]. All amplitude values were computed over the entire length of the study records (60 s). ISNR was computed twice, once after MECG subtraction from DREC (ISNR1) and one after IFECG denoising (ISNR2; Fig. (**1**)). Thus, ISNR2 actually describes the quality of the final IFECG tracing obtained from DREC using the SBMM.

### 2.4 Statistics

Distributions of DFECG amplitude, IFECG amplitude, DN amplitude, IN amplitude, DSNR and ISNR were described in terms of median [25^th^; 75^th^] percentiles and compared using the Wilcoxon Rank-Sum test for equal medians. Association between DFECG and IFECG (which are two different representations of the same electrophysiologic phenomenon, which is the electrical activity of the fetal heart) was evaluated using the Pearson’s correlation coefficient (ρ). Statistical significance level P was set at 0.05 in all cases.

## 3 RESULTS

By way of example, SBMM application to record 1 is depicted in Fig. (**2**), where simultaneously acquired DREC and IREC (channel 1) are represented together with all their components. As it can be seen, DFECG amplitude was much higher than IFECG amplitude (104 µV *vs.* 18 µV), whereas the amplitude difference between DN and IN was less marked (34 µV *vs.* 14 µV). Consequently, DSNR was higher than ISNR1 (4.9 dB *vs.* 1.0 dB). The noise level affecting IFECG at the end of the SBMM procedure was very low so that DSNR was lower than ISNR2 (4.9 dB *vs.* 11.2 dB).

Generalizing, for all records DFECG was always characterized by amplitude higher than that characterizing IFECG, independently of the channel (Table **1**). Consequently, median (over the records) DFECG amplitude was significantly higher than median IFECG amplitude (104 [89;157] µV *vs.* 22 [16, 28] µV, P=7.66·10^-4^). Instead, DN amplitude was higher or equal to IN amplitude (independently of channel) in records 1, 4 and 5, comparable in record 3 and lower in record 2 (Table **1**). Consequently, median DN amplitude and median IN amplitude were not significantly different (70 [39;78] µV *vs.* 49 [25;77] µV, P=0.45). Moreover, in the direct acquisition modality, median DFECG amplitude was significantly higher than median DN amplitude (P=3.17·10^-2^), whereas in the indirect acquisition modality, median IFECG amplitude was significantly lower than median IN amplitude (P=1.90·10^-3^). Thus, DSNR was greater than ISNR1 in all channels of every record but channels 1 and 2 of record 5 (Table **1**). Consequently, median DSNR was significantly greater than median ISNR1 (3.3 [1.6;4.8] dB *vs.* -2.3 [-7.4;0.6] dB, P=3.90·10^-3^). At the end of SBMM processing, however, the noise level affecting IFECG was mostly removed so that median DSNR was significantly lower than median ISNR2 (3.3 [1.5;4.8] dB *vs.* 9.6 [8.0; 10.9] dB, P=9.84·10^-4^).

Correlation between DFECG and IFECG was typically high and significant (ρ=0.78 [0.75;0.83], P<10^-208^; Table **1**). Only in records 3 and 5, ρ showed lower but still significant values (ρ=0.45 in channel 1 of record 3, and ρ=0.28 in channel 3 of record 5, respectively; P<10^-208^) in correspondence of the channel with the lowest ISNR1 (-11.1 dB and -1.8 dB, respectively; Table **1**).

Fig. (**3**) shows the 4 IREC channels of record 5 after subtraction of MECG (*i.e.* IFECG+IN). As it can be seen, represented signals show a significant amplitude variability among channels so that the IFECG component is more easily visible in some channels than in others. This finding can be generalized to all records. Indeed, ISNR1 variability among channels is a direct consequence of IFECG amplitude variability and IN amplitude variability (Table **1**).

## 4 DISCUSSION

This study evaluated SBMM suitability to denoise IREC in order to obtain an IFECG characterized by a signal quality at least comparable to that of DFECG, the latter being considered as the gold standard for FECG. Goodness of SBMM performance was assessed by correlating IFECG against DFECG. A good correlation between the two above-mentioned signals would justify the application of SBMM to IREC only, in future studies.

SBMM belongs to the class of template-based methods [11] for getting FECG. However, differently from the other proposed techniques, it introduces a modulation procedure to adjust for repolarization-length changes due to physiological heart-rate variability [29]. Thanks to this peculiar feature, SBMM strongly improves the accuracy of FECG estimation. A quantitative comparison of SBMM performance against other template-based methods with no modulation procedure is beyond the scope of the present work and was previously performed [28, 30]. However, a qualitative comparison may help understanding why SBMM represents an improvement with respect to the other template methods. IFECG estimation by template-based methods is obtained after MECG estimation and subtraction from IREC. If the modulation procedure is not applied, MECG is reconstructed as a tracing with fixed heart rate, so that in correspondence of the repolarization segment some misalignments may occur, causing significant artifacts in the resulting fetal tracing obtained by subtraction (Fig. (**4**), panel a). If the modulation procedure is introduced, maternal repolarization variability is tracked, and the artefacts are strongly reduced (Fig. (**4**), panel b). Analogously, when SBMM is applied for filtering fetal tracings (direct or indirect) from noise, it performs better than the other template-based techniques, since it is the only one able to track variations in fetal repolarization variability.

As every other template-based technique, SBMM works under the hypothesis of knowing R-peak positions. Here, to avoid confounding factors due to fetal R-peak misplacements, fetal R-peak detection was manually performed on DREC to ensure correct localization. Still, in order to evaluate the possible use of SBMM in real clinical cases in which DREC and IREC are typically not simultaneously available, it is important to observe that localization of the fetal R peaks (which must necessarily be automatic and not manual) is quite straightforward from DREC, where the DFECG component is dominant, whereas may become very challenging from IREC [31], where besides IFECG, are present other high amplitude components. Fetal R-peak localization is an interesting issue which, however, was beyond the scope of the present work but systematically treated by ourselves [17].

In our study, SBMM was applied to 60 s long recordings because the aim was the evaluation of a denoising technique for FECG applications and not the fetal parameters monitoring during labor, which typically last for hours. In studies interested in FECG-parameters monitoring during labor, 60 s windows of FECG should be recursively SBMM-filtered in order to get clean FECG tracings from which to perform measurements. Recursive application of SBMM allows almost real-time (one-minute delay) evaluation of such parameters and adaptation of the SBMM procedure to the physiological variability of FECG.

According to our results, DFECG amplitude was about an order of magnitude higher than IFECG amplitude (few hundred of µV the former, and tens of µV the latter) whereas the noise level was very variable over the records but, on average, comparable between the two acquisition modalities (direct *vs.* indirect). As a consequence, DSNR was usually higher than ISNR1. This finding matches expectations. DFECG is acquired with electrodes, which are in contact with the fetus, and, thus, its amplitude is expected to be quite large and DN is likely represented by fetal physiological signals other than FECG (such as fetal electroencephalography). Instead, IFECG is acquired by positioning the electrodes on the maternal abdomen, so that its amplitude is expected to be quite low and IN (which, according to our definitions does not include MECG) may incorporate both fetal and maternal physiological signals (such as fetal electroencephalography and maternal uterine contractions, *etc*). Despite the different signal amplitudes and the different levels of noise affecting them, DFECG and IFECG were strongly correlated (ρ=0.78), confirming that they are two different representations of the electrical activity of the fetal heart. The variability characterizing IFECG and IN amplitudes in the different channels is usually not reflected in the values of ρ, being correlation independent from signals amplitude. In addition, at the end of the SBMM procedure, ISNR2 was lower than DSNR, indicating that extracted IFECG was characterized by a better signal quality than DFECG.

A limitation of this study is the small number of records on which statistics were performed, due to the fact that because simultaneously recorded DREC and IREC are very rare. Nevertheless, the ρ values were statistically very significant in all cases (P < 10^-208^), so that the SBMM ability to correctly extract FECG from both DREC and IREC was clearly demonstrated in spite of the limited number of application cases. Only occasionally lower values of ρ were observed. These may indicate that electrodes have not correctly acquired the signal or that the transformation from DFECG to IFECG might not be perfectly linear, as assumed when computing ρ. This latter hypothesis is physiologically sustainable, since there is no determined geometrical relationship among the locations of the electrodes and the fetal heart.

## CONCLUSION

In conclusion, the high correlation observed between DFECG and IFECG indicates that these signals have equivalent morphological content; the finding that ISNR2 was higher than DSNR indicates that IFECG, obtained using SBMM, has better quality than DFECG. Thus, SBMM can be used to obtain clean, potentially clinically useful IFECG also when DFECG is not available. Thus, SBMM application to IFECG may contribute to the spread of this technique in the clinical practice, since able to provide good quality fetal tracings in a noninvasive, safe, simple and economic way. Future studies will compare clinical FECG parameters measured in DFECG and IFECG to confirm clinical utility of SBMM in clinical settings.

## Figures and Tables

**Fig. (1):**
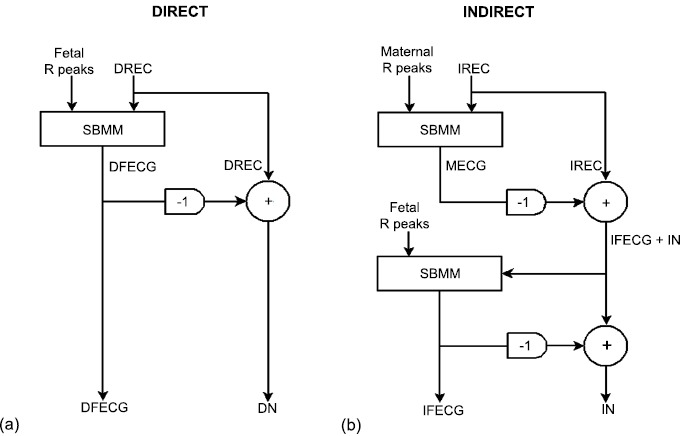
Block diagram of the procedure to extract DFECG and IFECG from DREC (panel a) and from a single channel of IREC (panel b), respectively, by means of SBMM.

**Fig. (2):**
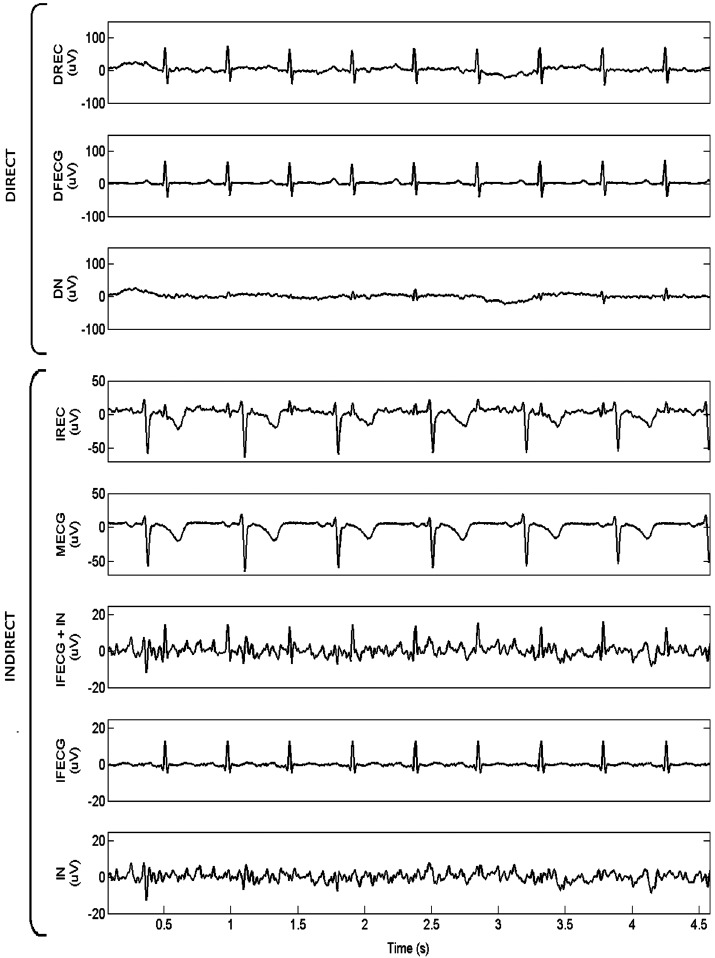
Representation of 4.5 s of simultaneously acquired DREC and IREC (channel 1) relative to record 1, together with all their components individually plotted (DFECG amplitude: 104 µV; DN amplitude: 34 µV; IFECG amplitude: 18 µV; and IN amplitude: 14 µV).

**Fig. (3):**
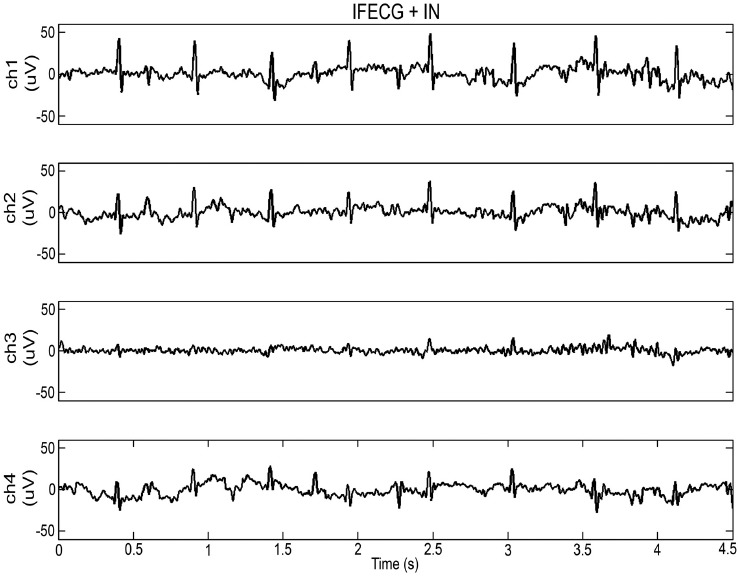
Representation of 4.5 s of the 4 IREC channels (Ch) of record 5 after subtraction of MECG.

**Fig. (4):**
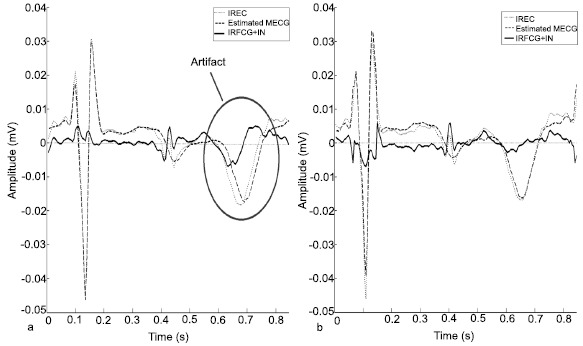
Example of a beat of the IFECG + IN (solid line) overlapped to estimated MECG (bold dotted line) and IREC (dotted line) obtained without (panel a) and with (panel b) the modulation/demodulation process.

**Fig. (A.1):**
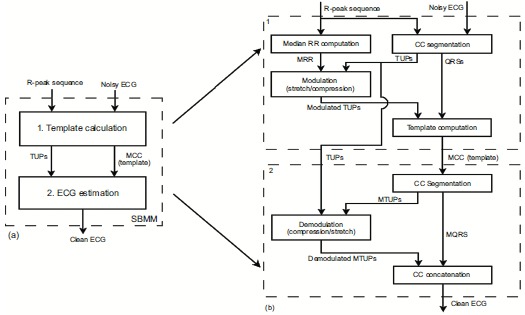
Block diagram of the SBMM procedure (panel a) and of the two consecutive steps of which it consists (panel b).

**Table 1:** Characteristics of the signal components in direct and indirect fetal recordings.

RCD	DREC			IREC					ρ
	DFECG ampl (mV)	DN ampl (mV)	DSNR (dB)	ch	IFECG ampl (mV)	IN ampl (mV)	ISNR1 (dB)	ISNR2(dB)	
1	104	34	4.9	1	18	14	1.0	11.2	0.85*
				2	23	30	-1.2	9.6	0.79*
				3	21	15	1.5	11.1	0.87*
				4	35	34	0.1	11.0	0.89*
2	87	41	3.3	1	5	72	-11.7	4.8	0.63*
				2	20	106	-7.3	9.6	0.82*
				3	13	94	-8.7	7.9	0.81*
				4	20	114	-7.5	9.1	0.75*
3	89	73	0.9	1	5	70	-11.1	6.0	0.45*
				2	18	77	-6.4	8.0	0.77*
				3	11	76	-8.4	7.9	0.75*
				4	22	83	-5.8	9.6	0.80*
4	208	70	4.8	1	24	56	-3.8	9.9	0.83*
				2	29	55	-2.8	10.0	0.76*
				3	25	31	-0.9	10.4	0.83*
				4	43	43	0	10.8	0.87*
5	140	94	1.8	1	54	26	3.2	11.7	0.77*
				2	41	24	2.3	10.9	0.76*
				3	10	16	-1.8	7.6	0.28*
				4	28	19	1.5	9.5	0.59*

*: P<10^-208^

## APPENDIX

### The Segmented-Beat Modulation Method (SBMM)

SBMM is an electrocardiographic (ECG) denoising procedure which can be applied when the positions of the R peaks are known [18-21]. It is based on the empirical observation that, in first approximation, the QRS-complex duration does not depend on the previous RR duration (*i.e.* on instantaneous heart rate) while the duration of the other ECG waves proportionally vary with it [28]. Consequently, each cardiac cycle (CC) can be segmented into QRS and TUP segments: the former, of fixed length, identified ±ΔT ms around the R peak; and the latter, of variable length, identified within ΔT ms after the R peak and ΔT ms before of the subsequent R peak. Thus, the QRS segment is 2∙ΔT ms long in all beats, while TUP segment duration is beat dependent and equal to the difference between CC duration and QRS duration.

The SBMM procedure consists of two consecutive steps (Fig. (**A.1**), panel a): 1. template computation; and 2. ECG estimation.

### A1 TEMPLATE COMPUTATION

A simplified (original detailed in [21]) block diagram of the template-computation step is depicted in the upper part of Fig. (**A.1**), panel b. The R-peak positions are used to identify all cardiac cycles (CCs) and to compute the median RR interval (MRR). After having segmented all CCs, the TUP segments are modulated (stretched or compressed) in order to have the length of the belonging CC to match MRR. Successively, a template beat (MCC) is obtained as the median of all modulated CCs reconstructed using the original QRS segments and all modulated TUP segments.

### A2 ECG ESTIMATION

A simplified (original detailed in [21]) block diagram of the ECG-estimation step is depicted in the lower part of Fig. (**A.1**), panel b. The clean ECG tracing is obtained by concatenating N times the template beat (N being the number of beats in the original noisy ECG recording) after demodulation (compression or stretching) of the TUP segments. Demodulation is performed in order to have estimated CC length equal to that of the corresponding CC in the original noisy recording. Optimization processes, involving cross-correlation maximization and distance minimization between the reconstructed and the original CCs, are included in the procedure to compensate small inter-beat, nonlinear heart-rate variations of the CC waveforms.

## LIST OF ABBREVIATIONS

Ch: = Channel
DFECG: = Direct fetal electrocardiogram
DN: = Noise affecting direct recordings
DREC: = Direct recording
DSNR: = Direct signal-to-noise ratio
ECG: = Electrocardiogram
FECG: = Fetal electrocardiogram
IFECG: = Indirect fetal electrocardiogram
IN: = Noise affecting indirect recordings
IREC: = Indirect recording
ISNR: = Indirect signal-to-noise ratio
ISNR1: = Indirect signal-to-noise ratio after subtraction of MECG from indirect recording
ISNR2: = Indirect signal-to-noise ratio after indirect recording denoising.
MCC: = Template beat
MECG: = Maternal electrocardiogram
MRR: = Mean RR-interval
RCD: = Record
SBMM: = Segmented-beat modulation method
SNR: = Signal-to-noise ratio
